# Cytokine profiles in acute liver injury—Results from the US Drug-Induced Liver Injury Network (DILIN) and the Acute Liver Failure Study Group

**DOI:** 10.1371/journal.pone.0206389

**Published:** 2018-10-25

**Authors:** Herbert L. Bonkovsky, Huiman X. Barnhart, David M. Foureau, Nury Steuerwald, William M. Lee, Jiezhun Gu, Robert J. Fontana, Paul J. Hayashi, Naga Chalasani, Victor M. Navarro, Joseph Odin, Andrew Stolz, Paul B. Watkins, Jose Serrano, for the US Drug-Induced Liver Injury Network and the Acute Liver Failure Study Group

**Affiliations:** 1 Department of Medicine, Wake Forest University School of Medicine, Winston-Salem, NC, United States of America; 2 Duke University School of Medicine, Durham, NC, United States of America; 3 Levine Cancer Center and Department of Research, Atrium Health, Charlotte, NC, United States of America; 4 Department of Internal Medicine, UT Southwestern Medical Center, Dallas, TX, United States of America; 5 Department of Medicine, University of Michigan, Ann Arbor, MI, United States of America; 6 Department of Medicine, University of North Carolina School of Medicine, Chapel Hill, NC, United States of America; 7 Department of Medicine, IUPUI, Indianapolis, IN, United States of America; 8 Department of Medicine, A Einstein Medical Center, Philadelphia, PA, United States of America; 9 Department of Medicine, Icahn School of Medicine at Mount Sinai Medical Center, New York, NY, United States of America; 10 Keck School of Medicine, University of Southern California, Los Angeles, CA, United States of America; 11 National Institute of Diabetes, Digestive, and Kidney Diseases, Bethesda, MD, United States of America; University of Navarra School of Medicine and Center for Applied Medical Research (CIMA), SPAIN

## Abstract

Changes in levels of cytokines and chemokines have been proposed as possible biomarkers of tissue injury, including liver injury due to drugs. Recently, in acute drug-induced liver injury (DILI), we showed that 19 of 27 immune analytes were differentially expressed and that disparate patterns of immune responses were evident. Lower values of serum albumin (< 2.8 g/dL) and lower levels of only four analytes, namely, IL-9, IL-17, PDGF-bb, and RANTES, were highly predictive of early death [accuracy = 96%]. The goals of this study were to assess levels of the same 27 immune analytes in larger numbers of subjects to learn whether the earlier findings would be confirmed in new and larger cohorts of subjects, compared with a new cohort of healthy controls. We studied 127 subjects with acute DILI enrolled into the US DILIN. We also studied 118 subjects with severe acute liver injury of diverse etiologies, enrolled into the ALF SG registry of subjects. Controls comprised 63 de-identified subjects with no history of liver disease and normal liver tests. Analytes associated with poor outcomes [death before 6 months, n = 32 of the total of 232 non-acetaminophen (Apap) subjects], were lower serum albumin [2.6 vs 3.0 g/dL] and RANTES [6,458 vs 8,999 pg/mL] but higher levels of IL-6 [41 vs 18], IL-8 [78 vs 48], and MELD scores [30 vs 24]. Similar patterns were observed for outcome of death/liver transplant within 6 months. A model that included only serum albumin < 2.8 g/dL and RANTES below its median value of 11,349 had 83% (or 81%) accuracy for predicting early death (or early death/liver transplant) in 127 subjects from DILIN. No patterns of serum immune analytes were reflective of the etiologies of acute liver failure, but there were cytokine patterns that predicted prognosis in both acute DILI and ALF.

## Introduction

Changes in levels of circulating cytokines and chemokines have been proposed as possible biomarkers of tissue injury, including liver injury due to drugs [1] [2]. Indeed, alterations in cytokine profiles have been reported in several experimental studies of DILI [3] [4]. In one study in humans, genetic polymorphisms associated with lower production of IL-10, an anti-inflammatory cytokine, were associated with lower eosinophil counts and poorer outcomes [2]. In an earlier paper from the US DILI Network (DILIN), we showed that, among 78 subjects with acute DILI, compared with 40 healthy controls, 19 of 27 immune analytes were differentially expressed and that disparate patterns of immune responses, especially those felt to be reflective of innate and adaptive cellular (mostly Th17) immunity were evident. Of particular note, relatively lower values at baseline of serum albumin (< 2.8 g/dL) in combination with relatively lower levels of only 4 specific analytes, namely, IL-9, IL-17, PDGF-bb, and RANTES, were highly predictive of early death, with positive predictive value [PPV] = 88%; negative predictive value [NPV] = 97%, and accuracy = 96% [1]. These and other recent results indicated that acute DILI is associated with varying, often robust immune responses and that relatively higher levels of expression of cytokines associated with innate immune activation are associated with poor prognosis, whereas higher levels of expression of cytokines associated with adaptive immune responses, like IL-17, portend good long-term prognosis with eventual recovery. Left unanswered by our earlier work was whether profiles of immune analytes in serum at onset might also be of value for distinguishing phenotypes or pathogenetic mechanisms of acute liver injury and/or ALF across more diverse causes. Similarly, in a recent report from the US Acute Liver Failure Study Group [ALF SG] that involved 39 subjects with idiosyncratic DILI, 21 with acetaminophen [Apap]-induced ALF, and 10 with ALF ascribed to acute viral hepatitis, increased levels of IL-17 were found in ~ 60% of subjects, regardless of etiology, as were levels of IL-21, which also are produced by Th17 cells [5].

The goals of the present study were to assess levels of 27 immune analytes in larger numbers of subjects enrolled in two national registry consortia, namely, those of the US Drug-Induced Liver Injury Network [DILIN] and of the Acute Liver Failure Study Group [ALFSG]. We were particularly interested to learn whether and to what extent the earlier findings from DILIN [1] would be confirmed in a new and larger cohort of 127 subjects with acute liver injury ascribed to drugs and chemicals, compared with a new cohort of 63 healthy controls. We also set out to assess profiles in subjects with more severe acute liver injury, enrolled into the ALF registry, with injuries ascribed to idiosyncratic DILI [not due to Apap], as well as to acute hepatitis B, to autoimmune hepatitis, or to Apap.

## Methods

The IRBs of all components of the US Drug-Induced Liver Injury Network and of the Acute Liver Failure Study Group approved this research. All subjects gave verbal and written consent to participate.

In this work, we studied subjects enrolled into two observational studies sponsored by the NIDDK/NIH, namely, DILIN and the ALF SG. The Drug Induced Liver Injury Network (DILIN) is a prospective, collaborative study of drug-induced liver injury in the United States, which was initiated in 2003 as a cooperative agreement funded by the National Institutes of Health (NIH)[6];[7] Adults and children greater than 2 years of age with documented clinically significant DILI as evidenced by any of the following criteria were included in the DILIN prospective study: (1) jaundice or serum bilirubin > 2.5 mg/dL, and any elevations in ALT, AST, or alkaline phosphatase, (2) no jaundice and serum bilirubin <2.5 mg/dL, but elevations in ALT or AST (> 5 x ULN on at least two occasions, at least 24 hours apart) or elevations in alkaline phosphatase (>2xULN on at least two similar occasions), (3) elevations in ALT or AST > 5x baseline values or elevations in alkaline phosphatase > 2x baseline values in persons with known pre-existing liver disease. Subjects were excluded for the following reasons: (1) other known causes of acute liver injury, such as acute cholangitis, acute viral hepatitis, or autoimmune liver disease, (2) Apap hepatotoxicity, (3) liver transplantation prior to the onset of DILI, (4) failure to give informed consent, or (5) inability or unwillingness to comply with case ascertainment procedures. Additional details are in Supplementary Material. Because our goal here was to study subjects with truly acute liver injury of diverse causes, we limited subjects to 127 who were enrolled within 14 days of the onset of symptoms and signs of DILI, and who eventually were adjudicated to have experienced definite, highly likely, or probable DILI [see below] and for whom adequate sera were available for analyses. These subjects were enrolled in the DILIN registry between Sep 1, 2004 and Oct 13, 2016; none was included in the previous paper [1]. Selected clinical, demographic, laboratory, and imaging data were collected at study entry, and blood samples were drawn, processed, and sera frozen at -80°C and sent on dry ice to the NIDDK sample repository, where they were stored until retrieved for analyses. We tried to restudy all subjects who recovered, after the acute DILI event at follow-up visits with repeat blood draws at ~6 months after the acute episode. If there was evidence of ongoing liver injury or abnormality at 6 months, we asked them to return again at 12 and 24 months after enrollment.

After at least 6 months of follow-up information was available, cases were adjudicated for the likelihood that the injury was due to the implicated drugs or HDS by a causality committee of experienced hepatologists [8]. All cases were scored as being definite (1: ≥95% likelihood), highly likely (2: 75–94%), probable (3: 50–74%), possible (4: 25–49%) or unlikely (5: <25%). For cases with more than one implicated agent, each drug or HDS was scored separately in a similar manner. For the purposes of this analysis, only cases scored as probable, highly likely or definite were used. All cases were also graded for severity on a scale of 1 to 5 as mild, moderate, moderate and hospitalized, severe or fatal using standardized criteria [9].

All deaths and liver transplants recorded in the DILIN Prospective study were assessed by a committee of DILIN in a standardized manner, and the role of the drug- or HDS- induced liver injury was scored as the primary cause, a contributory cause or not related [9].

Liver biopsies were not required as a part of the DILIN prospective protocol, but, if performed in the course of routine medical care, requests were made that de-identified, recut, unstained slides be prepared and sent to the Laboratory of Pathology, National Cancer Institute, in the NIH Clinical Center (Bethesda, MD). All such biopsies were read by the DILIN hepatic pathologist (D.E. Kleiner) without specific clinical information and scored for multiple findings, previously described [10].

### Subjects with acute liver failure

The Acute Liver Failure Study Group (ALFSG) has studied prospectively more than 2,700 cases of ALF over 20 years (1998-present) as well as ~770 fitting the definition of acute liver injury (ALI), defined as severe liver injury associated with INR ≥ 2.0 with no hepatic encephalopathy present. The criteria for ALF include INR ≥1.5 with any degree of hepatic encephalopathy after an acute hepatic illness occurring less than 24 weeks after onset of the acute disease. For all enrolled, ALFSG obtains clinical and demographic data; serum and DNA samples are also obtained from most of these subjects. Subjects from ALFSG included in the present study were those diagnosed as having acute idiosyncratic DILI (not due to Apap, n = 39) [11], patients with ALF ascribed to Apap (n = 13), to auto-immune hepatitis [AIH] (n = 38), to acute hepatitis B (n = 28). In the ALF study, informed consent was obtained from next of kin because patients by definition had altered mentation. Subjects with ALF were followed until the time of death or liver transplant or for up to 21 days after enrollment. Longer-term follow-up was generally not available for these subjects.

### Controls without evidence of any liver disease

De-identified subjects (n = 63) with no history of liver disease and normal hepatic function profiles (serum albumin, alanine aminotransferase, alkaline phosphatase, and total bilirubin) attending adult yearly well-patient follow-up clinic visits at Atrium Health (formerly Carolinas Health Care System, Charlotte, NC) practices were identified, and serum samples were collected, processed and stored, as described above.

### Immune analyte profiling by bio-plex assay

Concentrations of immune analytes in sera were determined using a human 27-plex assay (14 cytokines (IL-1beta, IL-1ra, IL-2, IL-4, IL-5, IL-6, IL-9, IL-10, IL-12, IL-13, IL-15, IL-17, IFN-gamma, TNF-alpha); 7 chemokines (Eotaxin, IL-8, IP-10, MCP-1, MIP-1-alpha, MIP-1beta, RANTES); and 6 growth factors (IL-7, FGF basic, G-CSF, GM-CSF, PDGF-BB, VEGF)) (Bio-Plex Suspension Array System, Bio-Rad, Hercules, CA, USA), following the manufacturer’s instructions. Samples were diluted 1:4 (v:v) in sample diluent and incubated for 30 minutes at room temperature, 300 rpm agitation with capture antibody-coupled magnetic beads. Following three washes in a Bio-Plex Pro wash station, samples were incubated for 30 minutes in the dark (room temperature, 300 rpm agitation) with biotinylated detection antibody. Each captured analyte was detected by the addition of streptavidin phycoerythrin and quantified using a Bio-Plex array reader. Analyte concentrations were calculated with Bio-Plex Manager software. Normal ranges for serum concentrations of immune analytes were based on measurements obtained from the healthy controls and recorded as mean +/-1 standard deviation (SD). Concentrations in sera of DILI or ALF subjects >1 SD higher or lower than the means of healthy subjects were defined as abnormal.

### Statistical methods

Descriptive statistics were used to describe the cohorts. These include means and standard deviations, median, 25^th^ and 75^th^ percentiles, minimum and maximum for continuous data, and counts with percentages for categorical data. For comparisons of continuous variables between groups, the Wilcoxon rank sum test for two groups or the Kruskal-Wallis test for more than two groups was employed. For comparisons of categorical data between groups, the chi-square or Fisher’s exact test (for small sample) was employed. Sensitivity, specificity, positive and negative predictive value and accuracy were calculated when predicting acute death. SAS version 9.4 (SAS, Inc, Cary, NC, USA) was used for all analyses. A two-tailed p-value of less than 0.05 was considered statistically significant.

The following modeling process was used to select variables among 27 immune analytes and routine clinical lab test results [serum albumin, total bilirubin] for prediction of early death [within 6 months of DILI onset] or early death/liver transplant for the DILIN data and the ALF data separately. Because DILIN didn’t enroll any subjects due to Apap, we restrict ALF data to those not due to Apap with n = 105 subjects in this process. To determine if the previous published model [1] can be confirmed with the new DILIN data or the ALF data, the same logistic regression models for predicting early death with the same variables were fitted to estimate the corresponding sensitivity, specificity and accuracy. In the event that the published model did not work well with these new data, a modeling process with variable selection was performed for the DILIN data and the ALF data, separately. Due to relatively small samples sizes for each etiology of acute liver disease and the relatively large number of variables studied, our goal was to find a stable, robust model with the smallest number of independent variables that are most predictive of prognosis. In the first step, we compared immune analytes between those died vs those survived or those with early death/liver transplant vs. other to select potential predictors. Those immune analytes that were statistically significant at p ≤ 0.05 level were considered in the second step in the stepwise logistic regression. If the number of potential predictors is less than the number of events, then all these potential predictors were considered in the second step. If the number of potential predictors is larger than the number of events, pairwise correlations between these predictors were examined. For those pairs of variables with high correlation, only the predictor with smaller p-value in predicting death was retained in the second step. The linear combination of these variables in continuous scale may not be the best predictor for early poor outcome because the values of the immune analytes can tend to be skewed. In the second step, all potential immune analytes were dichotomized at their observed median values. The dichotomized immune analytes were then used in the multivariate logistic regression with forward, backward and stepwise variable selection process. Once the final set of variables had been selected, the area under curve (AUC) was estimated for its potential prognostic and diagnostic value. A logistic regression model with all of these variables was fit to estimate the AUC based on the fitted model with linear combination of the binary variables as a predictor. Two summary binary variables, namely, serum albumin (≤2.8 g/dL or not, as used in our first publication [1]) and MELD score at baseline (≤ 17 or not), were considered in the logistical modeling. The following four different models were used for presentation: with serum albumin only, with MELD score only, with selected binary immune analytes only, with a combined binary of serum albumin and immune analytes. We expect that the binary variable, based on both immune analytes and clinical lab data, to have the highest predictability for early poor outcome. This binary variable has a value of 1 if values of the variables (immune analytes and clinical lab values) all fall in the binary category that was predictive of early death based on direction of association, and a value of 0 otherwise. The predictability of this binary variable for early poor outcome was evaluated by positive predictive value (PPV), negative predictive value (NPV) and accuracy (percent of correct prediction). Sensitivity and specificity were also calculated.

## Results

### Subjects studied with liver injury

We studied 127 subjects with acute DILI enrolled into the US DILIN, all of whom were enrolled within 14 days of the onset of symptoms and signs of DILI and all of whom were assessed to have definite, highly likely, or probable acute DILI. None of these subjects had been included in our previous publication [1]. The causative agents were many and varied, as summarized in **S3 Table**. They were in keeping with results previously reported for the entire DILIN cohort [7]; [12]. We also studied a total of 118 subjects with severe acute liver injury of diverse etiologies, enrolled into the ALF SG registry of subjects. The causes of ALF in the latter subjects were Apap in 13, drugs other than Apap in 39 [known causative agents summarized in **S4 Table**], severe autoimmune hepatitis (AIH) in 38, and severe viral hepatitis B in 28. Selected demographic, laboratory, and outcome data on the subjects studied are summarized in **Table 1.** As one might have anticipated, subjects with ALF due to Apap were younger and more often women. Also striking is that African-Americans accounted for more than 35% of cases of ALF due to all causes other than Apap (more than twice the percentage among the less severe DILIN-acute cases (17.3%)), but accounted for none of the ALF-Apap cases. This trend is consistent with another recent finding, namely, that the severity of DILI is greater in African-Americans than in other racial groups [13].

**Table 1:** Selected demographic, lab, and clinical features of subjects studied.

	DILIN Acute	ALF-Drug, not Apap	ALF-AIH	ALF-Hep B	ALF-Apap
Characteristic	N = 127	N = 39	N = 38	N = 28	N = 13
**Age** (years)					
N	127	39	38	28	13
Mean (SD)	47 (17.8)	48 (15.5)	53 (16.7)	43 (12.4)	36 (12.7)
Median (Q1, Q3)	50 (33, 61)	49 (35, 60)	57 (43, 67)	44 (34, 54)	33 (29, 42)
Min, Max	7, 77	17, 73	21, 78	20, 69	18, 66
**Female** (#/total (%))	70/127 (55.1%)	23/39 (59.0%)	28/38 (73.7%)	14/28 (50.0%)	8/13 (61.5%)
**Race/** **Ethnicity**					
White	94/127 (74.0%)	25/39 (64.1%)	24/38 (63.2%)	16/28 (57.1%)	13/13 (100.0%)
Black	22/127 (17.3%)	14/39 (35.9%)	14/38 (36.8%)	12/28 (42.9%)	0/13 (0%)
Other	11/127 (8.7%)	0/39 (0%)	0/38 (0%)	0/28 (0%)	0/13 (0%)
**BMI** (kg/m^2^)					
N	122	33	33	22	13
Mean (SD)	28.3 (7.17)	30.2 (8.49)	34.0 (12.18)	28.3 (6.18)	27.1 (6.12)
Median (Q1, Q3)	27.3 (23.7, 31.4)	27.1 (24.1, 34.8)	32.4 (26.0, 37.1)	25.8 (24.5, 30.7)	26.9 (23.2, 30.7)
Min, Max	17.4, 61.1	18.7, 53.7	19.1, 78.1	16.9, 43.1	18.1, 41.5
**ALT** (IU/L)					
N	127	38	36	27	12
Mean (SD)	1084 (1493)	909 (1182)	581 (729)	2069 (2391)	3769 (1798)
Median (Q1, Q3)	551 (204, 1321)	322 (225, 1025)	282 (148, 699)	1207 (292, 2176)	3565 (2686, 5352)
Min, Max	23.0, 9108	11.0, 4890	29.0, 3687	87.0, 9150	687, 6558
**AST** (IU/L)					
N	123	39	36	27	13
Mean (SD)	1005 (1660)	1024 (1557)	707 (773)	2165 (2978)	4916 (4127)
Median (Q1, Q3)	476 (134, 1058)	426 (176, 1065)	363 (178.5, 920)	576 (224, 2743)	4120 (2070, 6962)
Min, Max	32, 10,920	59, 7,373	42, 2,817	56, 9,901	161, 14,580
**AP** (IU/L)					
N	122	38	35	25	13
Mean (SD)	291 (406)	310 (361)	175 (81)	154.(51.1)	148 (46.9)
Median (Q1, Q3)	211 (145, 334)	190 (127, 311)	157 (121, 210)	140 (119, 179)	144 (103, 185)
Min, Max	38, 4148	45, 1679	92, 460	91, 294	92, 218
**STBR** (mg/dL)					
N	127	39	36	27	13
Mean (SD)	8.3 (7.4)	23.8 (12.3)	25.0 (8.9)	18.8 (9.7)	5.4 (3.5)
Median (Q1, Q3)	6.0 (2.7, 12.4)	22.8 (15.8, 30.3)	25.3 (19.8, 29.9)	18.5 (10.5, 24.3)	4.0 (3.4, 7.5)
Min, Max	0.1, 32.3	1.4, 52.2	6.8, 47.2	3.6, 42.0	2.2, 14.5
**INR** at baseline					
N	123	39	38	28	13
Mean (SD)	1.5 (0.89)	3.0 (2.35)	3.7 (4.04)	4.0 (2.52)	3.7 (2.38)
Median (Q1, Q3)	1.1 (1.0, 1.6)	2.4 (1.8, 3.0)	2.7 (2.0, 3.5)	2.7 (2.2, 5.9)	2.9 (1.8, 5.0)
Min, Max	0.9, 5.4	1.4, 11.3	1.6, 26.1	1.2, 9.7	1.6, 8.2
**Hemoglobin** (g/dL)					
N	121	38	36	28	12
Mean (SD)	13.1 (2.26)	11.3 (2.70)	11.6 (2.12)	11.4 (2.15)	10.8 (2.54)
Median (Q1, Q3)	13.4 (11.2, 14.6)	11.3 (9.7, 13.2)	11.6 (10.0, 13.1)	11.0 (10.2, 12.3)	10.4 (8.5, 12.4)
Min, Max	8.3, 20.6	1.6, 16.2	6.8, 17.0	7.8, 18.0	7.7, 15.3
**WBC** (x10^-3/^uL)					
N	122	39	37	28	13
Mean (SD)	7.7 (4.48)	11.7 (6.14)	15.4 (13.74)	11.6 (5.00)	7.8 (3.78)
Median (Q1, Q3)	6.8 (5.1, 8.9)	10.8 (6.8, 15.4)	10.9 (7.4, 17.2)	10.8 (8.7, 15.1)	8.4 (6.4, 8.7)
Min, Max	0.4, 39.6	0.3, 27.0	1.1, 63.8	2.5, 24.9	1.3, 16.3
**Platelet count** (x10^-3/^uL)					
N	122	39	37	28	13
Mean (SD)	228 (109)	160 (92)	143 (96)	143 (77)	126 (113)
Median (Q1, Q3)	211 (159, 272)	139 (88, 221)	118 (80, 180)	141 (92, 196)	88 (68, 124)
Min, Max	7.0, 640	26.0, 376	18.0, 472	30.0, 326	14.0, 427
**Eosinophils** absolute (#/uL)					
N	92	28	27	19	10
Mean (SD)	182 (281)	316 (833)	114 (312)	85.8 (113)	47.2 (100)
Median (Q1, Q3)	72.9 (0.1, 212)	4.1 (0.0, 215)	0.0 (0.0, 82.0)	31.5 (0.0, 164)	0.0 (0.0, 55.0)
Min, Max	0.0, 1540	0.0, 3724	0.0, 1480	0.0, 447	0.0, 320
**Albumin** (g/dL)					
N	121	38	35	25	13
Mean (SD)	3.4 (0.81)	2.6 (0.70)	2.3 (0.55)	2.7 (0.52)	3.2 (0.65)
Median (Q1, Q3)	3.4 (2.9, 4.0)	2.5 (2.2, 2.8)	2.3 (1.9, 2.7)	2.7 (2.5, 3.0)	3.0 (2.7, 3.9)
Min, Max	0.6, 5.0	1.3, 4.5	1.1, 3.7	1.1, 3.5	2.1, 4.2
**Globulin** (g/dL)					
N	73	28	26	16	11
Mean (SD)	3.1 (1.17)	3.3 (0.98)	4.0 (1.17)	3.4 (1.20)	2.2 (0.38)
Median (Q1, Q3)	3.1 (2.5, 3.8)	3.4 (2.4, 3.9)	3.9 (3.1, 5.0)	3.0 (2.6, 3.9)	2.2 (2.0, 2.5)
Min, Max	0.5, 6.3	1.6, 5.3	1.6, 6.4	2.2, 6.4	1.4, 2.8
**MELD** score at baseline					
N	118	39	36	27	13
Mean (SD)	18 (8.4)	32 (6.6)	34 (5.8)	34 (6.6)	32 (7.5)
Median (Q1, Q3)	17 (12, 24)	32 (27, 37)	35 (30, 40)	34 (28, 40)	34 (27, 37)
Min, Max	6.0, 40	16, 40	21, 40	22, 40	20, 40
**Death** within 6 months	18/127 (14.2%)	2/39 (5.1%)	7/38 (18.4%)	5/28 (17.9%)	2/13 (15.4%)
**Liver Transplant**	7/127 (5.5%)	3/39 (7.7%)	10/38 (26.3%)	3/28 (10.7%)	1/13 (7.7%)
**Both liver transplant and death**	2/127 (1.6%)	0/39 (0.0%)	3/ 38 (7.9%)	1/28 (3.6%)	0/13 (0.0%)
**Liver transplant and/or death**	23/127 (18.1%)	5/39 (12.8%)	14/38 (36.8%)	7/28 (25%)	3/13 (23.1%)

MELD is model for end-stage liver disease.

As might have been anticipated, the levels of serum ALT and AST at study enrollment were higher among subjects with ALF due to Apap, whereas levels of STBR in this cohort were lower. Also striking were the lower platelet counts among subjects with ALF, regardless of etiology, compared with those with less severe acute DILI. Regardless of etiology or severity, median and mean values for absolute eosinophil counts were all less than 500/uL, emphasizing that the subjects generally did not have evidence of allergic or hypersensitivity reactions. In addition, as one might expect, median values of MELD scores (32–35) [model of end-stage liver disease] were significantly higher in those with ALF, regardless of etiology, than in those with acute DILI of lesser severity (17). Nonetheless, 18 (14.2%) of acute DILI subjects had died, among whom 2 had required liver transplants within 6 months of onset, and 5 additional DILIN subjects had required liver transplants and had survived for at least 6 months. Overall, 23/127 (18.1%) of subjects with acute DILI died and/or underwent liver transplant within 6 months of DILI onset, similar to the percentages of ALF-drug non-Apap subjects who died or received liver transplants within 6 months.

### Levels of immune analytes in subjects studied are summarized in Fig 1

In this work, we have adhered to the general categorization of analytes that we used previously [1]: “Innate cytokines” not requiring actions of T or B cells—IL-1b, IL-6, TNF alpha; “adaptive cellular cytokines”—those that enhance/support T cell functions—IL-12p70, IFN gamma, IL-2, IL-15, IL-17; “NFkB-dependent cytokines”—IL-8, MIP1a, MIP1b, MCP1; “cytokines involved in resolving inflammation”—IL-1ra, IL-10; “other chemokines”—IP-10, RANTES, eotaxin; and “growth factors”—IL-7, FGFb, GCSF, PDGFbb, and VEGF. As expected, values of many of the analytes were higher among subjects with acute liver injury than among the healthy controls, and values were often different among the several groups of subjects with liver injury. However, despite numerous sub-group analyses and comparisons, we did not unveil patterns of expression of immune analytes that were selective or specific for etiology of acute liver injury, nor that were selective for any of the several drug causes of the liver injury. Striking findings were as follows: 1. ALF-Apap subjects had very high levels of IL-6 and MCP-1 and low levels of IL-9; 2. ALF subjects with other etiologies had low levels of IL-4, IL-13, IL-17, RANTES, PDGF bb, and VEGF; 3. Acute DILI subjects had low levels of IL-2 and IL-15, but no changes in IL-5, compared to healthy controls. We did not observe any significant correlations of levels of serum analytes and gender, age, or ethnicity of subjects, nor, for those ascribed to DILI, of the causative drugs. However, as regards the latter, it is important to keep in mind that there were many and diverse drug causes, so that it would be unlikely that a single drug cause would show significant difference from all others.

**Fig 1:**
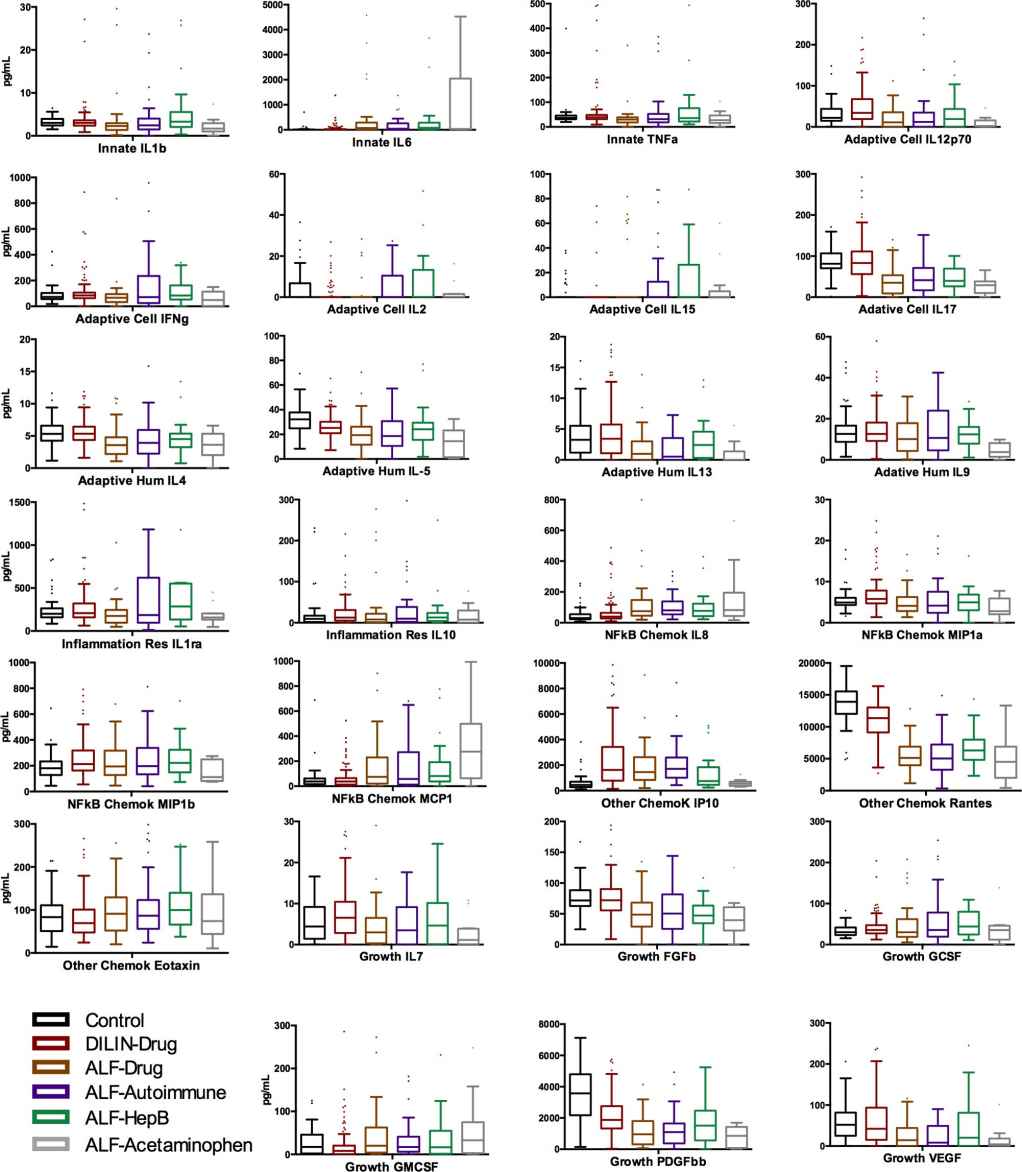
Box and whisker plots of immune analyte results for all subjects in this study.

### Analytes significantly correlated with severity of liver injury

In general, as we had observed before [1], subjects with more severe liver injury due to DILI had *lower* values of immune analytes than those with less severe acute injury, albeit higher than those of healthy controls. Levels of 20 immune analytes were significantly different in those with more severe acute DILI (severity scores 4–5) vs less severe acute DILI (severity scores 1–3). As described in our original work, plasma concentrations of 7/9 adaptive cytokines measured in our study, growth factors such as PDGF-bb and the chemokine RANTES were *lower*, whereas IL-6 was greatly elevated in the more severe group. In addition, as expected, median MELD scores (24 vs 15, P <0.001) and INR values (1.7 vs 1.0, P < 0.001) were higher in the more severely affected groups.

When we used MELD score at study entry as a marker of severity (MELD < 20 vs ≥ 20), among the subjects with acute DILI, we found a total of 7 immune analytes significantly different with 5/7 being *lower* in subjects with MELD ≥ 20, namely, IL-12, IL-17, PDGF-bb, RANTES, and TNFa, whereas IL-6 and IL-8 were *higher*. In addition, as was anticipated, the median level of serum albumin was lower in those with higher MELD scores (3.3 vs 3.7 g/dL, p = 0.009), whereas INR was higher (1.7 vs 1.1, p< 0.001).

In the acute DILI cohort, only three analytes were significantly associated with elevations of serum total bilirubin (STBR > 1.2 mg/dL, n = 105), namely, IL-8 (43 vs 28, P <0.001); MIP-1b (224 vs 169, p = 0.007); and TNFa (39 vs 49, p = 0.020). If the critical value of STBR is chosen as > 2.5 mg/dL vs ≤ 2.5 mg/dL, only levels of IL-8, IP-10, and MIP-1b were all significantly higher. If one chooses the critical value of STBR > 6 mg/dL, only levels of IL-8 and MIP-1b continue to show significantly higher values.

### Predictors of prognosis/outcomes

Among subjects from the DILIN cohort, immune analytes that, at baseline were significantly different and correlated with death at 6 months were IL-6 (41 vs 10, p <0.001); IL-8 (60 vs 38, p = 0.002); IL-17 (53 vs 88, p = 0.003), FGFb (56 vs 76, p = 0.03); and RANTES (6,441 vs 11,493, p< 0.001). Note that serum albumin also was significantly lower in those who died (2.6 vs 3.6 g/dL, p < 0001). As expected, the median MELD score among those who died (MELD = 31) was significantly higher than the median MELD score (MELD = 17) of those who survived. Similar patterns were observed for early outcome of death or liver transplant for subjects with acute DILI from the DILIN, levels of 3 immune analytes (IL-17, RANTES, FGF-basic) and albumin were significantly lower in those who died (n = 18) vs those who survived at least 6 months (n = 109) and, in keeping with our earlier observations [1], IL-6 and IL-8 were elevated (**Table 2**).

**Table 2:** Immune analytes significantly associated with death [before 6 months] among Subjects with acute drug-induced liver injury from the DILIN.

Analyte	Died [n = 18]	Survived [n = 109]	P
Albumin*	2.6 [0.6–4.0]	3.6 [1.5–5.0]	<0.001
RANTES*	6,441 [2729–12313]	11,493 [4417–16363]	<0.001
IL-17*	53 [3–259]	88 [4–326]	0.003
FGF basic	56 [9–213]	76 [14–375]	0.03
IL-6	41 [9–1378]	10 [3–382]	<0.001
IL-8	60 [18–487]	38 [10–482]	0.002
MELD	31 [14–40]	17 [6–40]	<0.001
Significantly correlated in 1^st^ study but not 2^nd^			
IL-9*	11 [2–170]	13 [0–482]	0.595
PDGF-bb*	1,702 [29–4779]]	1,918 110–5742]	0.113

Results are median values with ranges in parentheses.

* Denotes analytes also significantly predictive of early death in the first report [1]

Among subjects with ALF, if we include all known etiologies (ALF-Apap, ALF-drugs other than Apap, ALF-AIH, and ALF-acute hepatitis B), there were a total of 16 subjects who died within 6 months and 29 subjects who died or had liver transplants. Only IL-6 and IL-12 were significantly different (p < 0.05), with IL-6 being significantly *lower* in those who died (median = 36 vs 71), whereas IL-12 was significantly *higher* in those who died (median = 23 vs 11, p = 0.042). In addition, there were trends towards differences (p < 0.10) for IL-17, FGF-basic, RANTES, and VEGF, with median values for all *higher* in those who died. Rather surprisingly baseline levels of serum albumin were not different among these cohorts between those who died vs those who survived, and MELD scores were significantly *lower* in those who died (median 29 vs 35, p = 0.001). Because subjects with ALF due to Apap are rather different from the other cohorts (**Table 1**) and because Apap overdose is the prototypical intrinsic, predictable, dose-dependent hepatotoxic drug, not dependent in pathogenesis upon the host immune response to injury, we thought it best to exclude the ALF-Apap subjects from further analyses.

Several immune analytes were different when we compared the subjects with acute DILI to those from subjects with ALF (**Table 3**).

**Table 3:** Immune analytes significantly different between DILIN and ALF cohorts—AIH, acute hepatitis B, and drugs, not acetaminophen.

Analyte	Acute DILIN [n = 127]	ALF Groups—Drugs, not Apap, AIH, Hep B, [n = 105]	P Values
IL-1b	3 [1–507]	2 [0–59]	0.010
IL-4	5 [2–33]	4 [0–16]	<0.001
IL-5	25 [7–373]	20 [0–133]	<0.001
IL-6*	12 [3–1378]	62 [4–42,705]	<0.001
IL-7	7 [0–1229]	4 [0–396]	<0.001
IL-8*	40 [10–482]	77 [20–7,282]	<0.001
IL-12*	34 [0–2371]	12 [0–264]	<0.001
IL-13	3 [0–400]	1 [0–88]	<0.001
IL-15	0 [0–649]]	0 [0–245]	0.002
IL-17*	83 [3–326]	39 [0–353]	<0.001
Eotaxin	69 [24–1094]	91 [20–2153]	0.003
FGF basic	74 [9–375]	49 [0–728]	<0.001
GM-CSF	9 [0–1850]	18 [0–307]	<0.001
IFN-gamma	85 [0–7639]	72 [0–2347]	0.041
MCP-1	39 [0–2550]	65 [0–2312] <0.001	<0.001
MIP-1a	6 [1–66]	4 [0–134]	<0.001
PDGF-bb*	1,890 [29–5,742]	1,104 [0–5,246]	<0.001
RANTES*	11,349 [2729–16,363]	5,699 [348–14,897]	<0.001
TNF-alpha*	40 [9–10,289]	32 [0–952]	0.002
VEGF	42 [0–942]	12 [0–343]	<0.001

Results are median [range].

* When using MELD score cutoff value of 20 to dichotomize severity of liver damage across DILIN and ALF patients IL-12, IL-17, PDGF-bb, RANTES, and TNFa, remained significantly lower whereas IL-6 and IL-8 were elevated.

When we combined the acute DILIN subjects and the ALF subjects due to drugs other than Apap, to AIH, and to acute hepatitis B, we found that a total of 32 died within 6 months and 49 died or had liver transplant within 6 months. Similar to our results for the acute DILIN subjects alone (**Table 2**), the analytes that were significantly different between those who died vs. survived in these combined cohorts were serum albumin, IL-6, IL-8, and RANTES (**Table 4**). The median MELD score was also significantly higher in those who died or required liver transplantation than in those who survived (30 vs 24, p = 0.023). Similar analytes were noted in comparison between died or liver transplanted *vs* all other.

**Table 4:** Immune analytes significantly associated with acute death or urgent liver transplant among subjects with acute drug-induced liver injury in the US DILIN and with ALF within 6 months due to causes other than apap.

Variable	Died [n = 32]	Survived [n = 200]	P
IL-6 [pg/mL]	41 [9, 1,378]	18 [3, 42,705]	0.009
IL-8 [pg/mL]	78 [18,487]	48 [10, 7,282]	0.008
RANTES [pg/mL]	6,458 [1548, 14,897]	8,999 [348, 16,363]	0.056
Albumin [g/dL]	2.6 [0.6, 4.0]	3.0 [1.1, 5.0]	<0.001
MELD score	30 [14, 40]	24 [6, 40]	0.023

Results are median [range].

However, when ALF subjects (due to drugs other than Apap, to AIH, and to acute hepatitis B) were examined separately, there were a total of 14 died who within 6 months and 26 who died or had liver transplant. In them, we found different analytes than in DILIN subjects that were significantly or marginally significantly different between those died *vs* survived or between those died or required liver transplants *vs* others. These analytes for died *vs* survived were IL-2, IL-4, IL-5, IL-9, IL-13, IL-15, FBF basic. Therefore, modeling for predicting early death or early death or liver transplant was carried out separately for the DILIN cohort and ALF cohort.

### Best models for predicting survival vs death at 6 months

For DILIN subjects, we were not able to fit the previous model [1] because there were no subjects with serum albumin ≤2.8 g/dL and the four immune analytes below the specified values. This may be due to different subjects and/or batches of reagents that we used for assessing relative levels of immune analytes than those of our prior work. Our modeling process in the 127 DILIN subjects studied here showed that the only independent immune analyte is binary RANTES with cutoff at observed median of 11,349 pg/mL where, as before [1], *lower* RANTES is associated with death within 6 months. **Table 5** presents the modeling results from four different logistical models with serum albumin alone, MELD score alone, selected binary RANTES alone, and combined binary variable of RANTES and serum albumin. The only independent and significant predictors of death within 6 months of DILI onset were serum albumin ≤2.8 g/dL and RANTES, elevated above those of normal controls, but relatively lower values, less than the median value (**Table 5**). The results are thus similar to the main results of our first paper [1], although that model used serum albumin and four immune analytes (IL-9, IL-17, PDGFbb, and RANTES), all of which were relatively lower in those who died than in those who survived. In the current acute DILIN subjects, lower serum albumin, ≤ 2.8 g/dL, is very nearly as good a predictor as the combination of lower serum albumin + lower RANTES, with overall accuracy 82% [95% CI 75–89%] vs 83% [95% CI 77–90%]. Both are notably superior to MELD score (less *vs* greater than its median value of 17), which had overall accuracy of only 64% (95% CI = 55–72%) (**Table 5**). The models for outcomes of either early death or liver transplant have similar overall accuracies.

**Table 5:** Models predictive of prognosis of acute liver injury among subjects with acute DILI from the US DILIN—death at 6 months vs survived.

Variable	Acute Death	Survived	Sensitivity (95% CI)	Specificity (95% CI)	Positive Predictive Value (95% CI)	Negative Predictive Value (95% CI)	Accuracy (95% CI)
Serum albumin							
< = 2.8 g/dL	10	15	56% (47%, 64%)	86% (80%, 92%)	40% (31%, 49%)	92% (87%, 97%)	82% (75%, 89%)
>2.8 g/dL	8	94					
MELD score at baseline (+/- 7 days)*							
≥17	16	42	94% (90%, 98%)	58% (50%, 67%)	28% (20%, 36%)	98% (96%,100)	64% (55%, 72%)
<17	1	59					
RANTES [pg/mL] below median	15	49	83% (77%, 90%)	55% (46%, 64%)	23% (16%, 31%)	95% (92%, 99%)	59% (51%, 68%)
RANTES above median	3	60					
RANTES and serum albumin							
RANTES below median and albumin ≤2.8 g/dL	7	10	39% (30%, 47%)	91% (86%, 96%)	41% (33%, 50%)	90% (85%, 95%)	83% (77%, 90%)
RANTES above median or albumin >2.8 g/dL	11	99					

Median value for RANTES was 11,349 pg/mL

*Some subjects had missing MELD score.

For all ALF subjects excluding ALF-Apap, the accuracy of serum albumin ≤ 2.8 g/dL in predicting eventual outcomes was appreciably lower, only 35%. Despite the weaker performance of this combination in the ALF subjects, they were notably better than MELD scores, in that the median MELD score was significantly *lower* in those who died than in those who survived within 6 months (29 vs 35, p = 0.001). For the best model for predicting acute death for subjects from the ALF Study, a combination of serum albumin < 2.8 g/dL and IL-13 greater than the median value of 2.73 pg/mL provided an accuracy of 73% [**Table 6**]. In this cohort, if the model in **Table 5** is used with the observed RANTES median value of 5,699 pg/dl and serum albumin less than or equal to 2.8 g/dL + RANTES greater than the median, the accuracy in predicting outcome at 22 days was not as good at 66%. The models for predicting acute death or liver transplant in the ALF subjects excluding ALF-Apap were somewhat different (**Table 7**) where IL-5 rather than IL-13 was selected in the final model although the accuracies were similar as the models for predicting acute death.

**Table 6:** Models predictive of prognosis of acute liver injury—subjects with acute liver failure due to drugs other than apap, to autoimmune hepatitis, or to acute hepatitis B–death at 6 months vs survived.

Variable	Acute Death	Survived*	Sensitivity (95% CI)	Specificity (95% CI)	Positive Predictive Value (95% CI)	Negative Predictive Value (95% CI)	Accuracy (95% CI)
Serum albumin							
≤2.8 g/dL	13	63	93% (88%, 98%)	25% (16%, 34%)	17% (10%, 25%)	95% (91%, 100%)	35% (26%, 45%)
>2.8 g/dL	1	21					
MELD score at baseline (+/- 7 days)							
≥ = 17	14	87	100% (100%, 100%)	1% (0%, 3%)	14% (7%, 21%)	100% (100%, 100%)	15% (8%, 22%)
<17	0	1					
IL-13 above median	10	42	71% (63%, 80%)	54% (44%, 63%)	19% (12%, 27%)	92% (87%, 98%)	56% (47%, 66%)
IL-13 below median	4	49					
IL-13 and serum albumin							
IL-13 above median and albumin ≤2.8 g/dL	9	23	64% (55%, 73%)	75% (66%, 83%)	28% (20%, 37%)	93% (88%, 98%)	73% (65%, 82%)
IL-13 below median or albumin >2.8 g/dL	5	68					

Median value for IL-13 = 1.27 pg/mL

* Total N for survivors not always 91 because of missing data to serum albumin and MELD score in a few subjects.

**Table 7:** Models predictive of prognosis of acute liver injury—Subjects with acute liver failure due to drugs other than apap, to autoimmune hepatitis, or to acute hepatitis B—death or liver transplant at 6 months vs survived without liver transplant.

Variable	Acute Death or Liver Tanspl.	Other*	Sensitivity (95% CI)	Specificity (95% CI)	Positive Predictive Value (95% CI)	Negative Predictive Value (95% CI)	Accuracy (95% CI)
Serum albumin							
< = 2.8 g/dL	23	53	92% (87%,97%)	27% (19%,36%)	30% (21%,39%)	91% (85%,97%)	44% (34%, 54%)
>2.8 g/dL	2	20					
MELD score at baseline (+/- 7 days)							
≥17	25	76	100% (100%, 100%)	1% (0%, 3%)	25% (16%,33%)	100% (100%, 100%)	25% (17%, 34%)
<17	0	1					
IL-5 above median	18	34	69% (60%, 78%)	57% (47%,66%)	35% (26%,44%)	85% (78%,92%)	60% (51%, 69%)
IL-13 below median	8	45					
IL-5 and serum albumin							
IL-5 above median and albumin ≤2.8 g/dL	15	24	58% (48%, 67%)	70% (61%, 78%)	38% (29%, 48%)	83% (76%, 90%)	67% (58%, 76%)
IL-13 below median or albumin >2.8 g/dL	5	67					

Median value for IL-5 = 9.81 pg/mL

* Total N for others not always 77 because of missing data to serum albumin and MELD score in a few subjects.

When we combined subjects with acute DILI from the US DILIN cohort and those with ALF due to drugs other than Apap, to AIH, or to acute hepatitis B [which groups are biologically and patho-physiologically more similar to the acute DILIN subjects], we found a total of three analytes that were significantly different between those who died vs those who lived, namely, serum albumin, IL-6 and IL-8, and one analyte that showed a strong trend towards significance, namely, RANTES (p = 0.056). The median MELD score of 30 [range 14–40] also was significantly higher in those who died than in those who survived (MELD = 24, [range 6–40], p = 0.023).

## Discussion

In this work, we report results of profiles of immune analytes in relatively large and diverse cohorts of subjects with acute liver injury due to DILI as well as to several other causes of acute liver failure. Our main findings are that levels of immune analytes are often changed in acute liver injury/ALF, compared with controls without liver disease. Despite our performing many statistical comparisons, we were not able to identify any unique or distinctive patterns of abnormalities that were predictive of or significantly or uniquely correlated with etiologies of ALF, nor with differing patterns of acute DILI–hepatocellular, *vs* cholestatic, *vs* mixed. We also were unable to find any patterns that were unique or selective for specific drug causes of acute DILI. However, because of the large number of incriminated drugs, each represented by only one or a few examples, we had little statistical power to unveil such patterns. We had hoped that profiles of immune analytes might help to improve further the reliability of RUCAM or DILIN methods for assessing causality in drug-induced or other forms of liver injury [14].

Most cytokine levels also were not correlated meaningfully with severity or outcomes of acute DILI or other etiologies of ALF. However, as in our earlier report [1], we again found that lower levels of serum albumin (< 2.8 g/dL, the critical value that defines Child-Turcotte-Pugh class C chronic liver disease) and RANTES (< median value of all samples run in the batch assay) at baseline were significantly predictive of those who were less likely to survive for at least 6 months (in subjects with acute DILI without acute liver failure).

As regards profiles of immune analytes in subjects with Apap DILI, we found high levels of IL-6 and MCP-1, whereas levels of IL-9 were low. Our assay platform, unfortunately, did not include IL-18, which recently has been singled out as important in Apap DILI in a murine model [15]. However, in our study, levels of IFN gamma in Apap DILI were not notably increased (**Fig 1**). This latter finding is not in keeping with recent results from the murine model of Apap DILI, in which prominent roles of IL-18 and one of its down-stream effectors, IFN gamma, were proposed [15]. Others showed that Apap decreased the mRNA expression of IL-1beta, IL-18, and the NLRP3 inflammasome complex in piglets [16].

The search for newer biomarkers, or groups of biomarkers that, at baseline, predict occurrence and eventual outcomes of acute liver injury thus continues. Additional recent candidate biomarkers include microRNA-122 (miR-122), glutamate dehydrogenase, total keratin 18, caspase-cleaved keratin 18, glutathione S-transferase alpha, alpha fetoprotein, arginase-1, osteopontin, sorbitol dehydrogenase, liver fatty acid binding protein, cadherin-5, macrophage colony stimulating factor receptor, paraoxonase 1 (normalized to prothrombin protein), and leucocyte cell-derived chemotaxin-2 [17]. In this recent work, glutamate dehydrogenase correlated more closely with serum ALT; levels of miR-122 were widely variable, both within and among subjects. The most useful analytes predictive of death within 6 months of onset were serum keratin 18, osteopontin, and macrophage colony stimulating factor receptor.

The extent to which miR-122 or other miRNA levels may improve our predictive algorithms remains to be ascertained and will be the subject of a later paper in this series.

Our models for predicting outcomes of acute DILI or ALF that included only levels of serum albumin and selected cytokines performed appreciably better overall than the MELD score (**Table 5A, 5B and 5C**). Perhaps, routine measurements of RANTES and IL-13, in addition to levels of serum albumin, will prove to provide a better model of prognosis of acute liver injury. Measurements of serum albumin are readily and widely available, whereas those of RANTES, IL-13, or other immune analytes are not. These would likely require quantification by enzyme-linked immunosorbent assays, which are more costly and involved than assays of albumin or the components used for calculation of MELD. However, the MELD score is now well accepted and widely available, so that additional confirmatory work with larger numbers of subjects would be required.

Undoubtedly, numerous factors influence levels of immune analytes in the serum, particularly in subjects with acute DILI or acute liver injury due to other causes. These may include other underlying diseases, acute sepsis or other illnesses, for which drugs have been given, as well as genetic or acquired host-specific factors (gender, age, ethnicity, etc.).

A finding in both our first paper [1] and in this new work is that levels of RANTES are lower in subjects with acute idiosyncratic DILI who are destined to have poor outcomes. RANTES (also known as C Chemokine Ligand-5, CCL5) is an integral component of liver inflammation and repair pathways. It is up-regulated in livers of patients with hepatitis C or alcoholic liver diseases and mediates in part the pathogenesis of experimental models of non-alcohol-induced liver steatosis or fibrosis [18]. RANTES is produced by the reticuloendothelial system of damaged liver tissue [19] and promotes chemo-attraction of myeloid cells and activation of Kupffer cells. While recruitment of inflammatory macrophages may contribute to liver injury [20], hepatic macrophages are a highly dynamic and complex network sensing altered tissue integrity and contributing in a major way to the maintenance of tissue homeostasis [21]. Kupffer cells, for instance, contribute T cell tolerance by, *inter alia*, expressing immune checkpoint agonist PD-L1, secreting prostaglandin PGE2 and indoleamine-2,3-dioxygenase. Recruitment of monocytes to the liver in case of injury is also an important prerequisite for liver regeneration and repair, as they help to clean up and to resolve liver injury and necrosis, and they also cross-talk with hepatic pericytes [22, 23]. Thus, we speculate that lower levels of RANTES foreshadow rather less robust macrophage functions and responses to acute DILI and portend poorer outcomes.

The current study has strengths that include sizable numbers of subjects, especially subjects from US DILIN. These subjects have been phenotyped in detail and have been followed for at least 6 months—or until they died or underwent LT. Alternative causes of acute liver injury were sought and reasonably ruled-out, and causality assessments were performed by a formal method, previously studied and found to be more reliable than simply RUCAM [14]. Subjects from ALF SG also were carefully characterized in a formal way, although, regrettably, due to limitations of resources, follow-up was generally followed for longer than 22 days (or until early death or LT). Nevertheless, we have performed analyte and data analyses on samples from the largest numbers of alternative diagnoses to acute idiosyncratic DILI, such as acute hepatitis B, AIH, as well as DILI due to Apap thus far assembled.

Limitations of this study include the following: Because of the characteristics of the reagents and methods for assessing relative levels of immune analytes, we are unable to combine the raw results of our previously published work [1] with the newer results summarized herein. Would we have obtained greater reproducibility and comparability between studies with ELISA assays for selected analytes, such as IL-9, IL-17, or RANTES? We do not know. In addition, although our total number of subjects with acute DILI was relatively high, the diverse drug causes (**S3 Table, and S4 Table**) led to too few subjects with DILI due to a single agent (except for ALF-Apap) to be able to draw any conclusions about possible drug-specific alterations in profiles.

Then, too, variability in severity and course of acute DILI/ALF in genetically diverse populations and insufficient numbers of non-European-Americans (African-Americans, Asians, or Hispanics) limit statistical power of sub-group analyses. With regard to DILI in African-Americans, it is striking that African-Americans accounted for more than 35% of cases of ALF due to all causes other than Apap (more than twice the percentage among the less severe DILIN-acute cases (17.3%)), but accounted for none of the ALF-Apap cases. This trend is consistent with another recent finding, namely, that the severity of DILI is greater in African-Americans than in other racial groups [13], suggesting that additional genetic or environmental factors important in modulating the severity of DILI still remain to be identified. These results also emphasize the importance of continued and expanded enrollment of persons of African descent into the US DILIN, the ALF SG, and other registries around the world.

Another limitation is that, although subjects from both DILIN and ALF SG were enrolled soon after the onset of liver injury (within 14 days), there was still some variability in the timing of obtaining the blood samples. In addition, due to the limitations of study designs, we did not have repeated samples obtained and measured during the acute phase of injury. However, in our earlier study [1], we did not find any significant differences in levels of immune analytes whether samples were collected between 1–14 days or 15–30 days of onset of injury. To address some of these limitations, in the ongoing US DILIN study, we are collecting repeated samples during the acute phase of injury in new cohorts of subjects.

## Supporting information

S1 Table More detailed description of the drug-induced liver injury network protocol: Clinical centers participating in DILIN since its inception in 2003. (PDF)

S2 Table More detailed description of the acute liver failure study group protocol and consortium: Clinical centers currently participating in the acute liver failure study group. (DOCX)

S3 Table Causative agents of acute drug-induced liver injury among subjects from the DILI. (DOCX)

S4 Table Causative agents of acute drug-induced liver injury among subjects from the acute liver failure registry. (PDF)

## Abbreviations

Alk P: serum alkaline phosphatase
ALT: serum alanine aminotransferase
ANA: serum anti-nuclear antibodies
Apap: acetaminophen
AST: serum aspartate aminotransferase
CK: cytokeratin
DCRI: Duke Clinical Research Institute
DILI[N]: Drug Induced Liver Injury [Network]
DRESS: drug rash with eosinophilia and systemic signs
ELISA: enzyme-linked immunosorbent assay
HAI: histology activity index
HDS: herbal and dietary supplements
INR: international normalized ratio
LT: liver transplantation
NCI: National Cancer Institute
NIH: National Institutes of Health
NIDDK: National Institute of Diabetes and Digestive and Kidney Diseases
PA: portal area[s]
R: the ratio of serum ALT/ULN for ALT divided by serum Alk P/ ULN for Alk P
SMA: serum anti-smooth muscle antibodies
STBR: serum total bilirubin
ULN: upper limit of normal
VBDS: vanishing bile duct syndrome
