# Correction: Cytokine profiles in acute liver injury—Results from the US Drug-Induced Liver Injury Network (DILIN) and the Acute Liver Failure Study Group

**DOI:** 10.1371/journal.pone.0212394

**Published:** 2019-02-11

**Authors:** Herbert L. Bonkovsky, Huiman X. Barnhart, David M. Foureau, Nury Steuerwald, William M. Lee, Jiezhun Gu, Robert J. Fontana, Paul H. Hayashi, Naga Chalasani, Victor M. Navarro, Joseph Odin, Andrew Stolz, Paul B. Watkins, Jose Serrano, for the US Drug-Induced Liver Injury Network and the Acute Liver Failure Study Group

The eighth author’s name is spelled incorrectly. The correct name is: Paul H. Hayashi.
