# Supplementary material for: Cytokine profiles in acute liver injury—Results from the US Drug-Induced Liver Injury Network (DILIN) and the Acute Liver Failure Study Group
Source: PLoS One. 2018 Oct 25;13(10):e0206389. doi: 10.1371/journal.pone.0206389 (PMC6201986; doi:10.1371/journal.pone.0206389)
Supplement: S1 Table — (PDF) [file pone.0206389.s001.pdf]

S1. More Detailed Description of the Drug-Induced Liver Injury Network Protocol

The Network has consisted of multiple clinical centers, a central data coordinating center and a sample and data repository maintained by the National Institute of Diabetes and Digestive and Kidney Diseases (NIDDK). The principal investigators and locations of the clinical centers have varied over the course of the study and are provided in **S1 Table**. The Duke Clinical Research Institute (DCRI: Durham, NC) has served as the data-coordinating center since the inception of DILIN. The DILIN Prospective Protocol allowed the clinical centers to enroll all patients with liver injury suspected to be due to drugs or herbal and dietary supplements (HDS) seen at their institutions within six months of onset who agreed to participate. Extensive demographic, clinical, laboratory, imaging and histologic material was retrieved from the patients' medical records, and a medical histories and physical examinations were performed at the time of enrollment. In addition, serum, plasma and whole blood were taken to perform laboratory tests that might not have been done initially and to provide samples stored at the NIDDK repository. Patients were asked to return in six months and, if liver tests, physical examination or imaging studies remained abnormal, again at 12 and 24 months after enrollment. All data were entered into an electronic database maintained at the DCRI. A more nearly complete description of the Prospective Protocol has been published[13] and the full text is available on the DILIN public website at: www.DILIN.org.

All details of the Prospective Protocol and consent forms were approved by local Institutional Review Boards as well as by a central Data Safety Monitoring Board established for this study by the NIDDK. All patients provided written informed consent.

S1 Table Clinical Centers Participating in DILIN since its Inception in 2003

University of Connecticut Health Center [2003-2008]: Drs. Herbert Bonkovsky (PI) and James Freston, Robert Rosson, and George Wu

University of North Carolina Chapel Hill [2003-2017]: Drs. Paul Watkins (PI) and Paul Hayashi. Subsites at Carolinas HealthCare System [2007-2014] and Wake Forest/NC Baptist Medical Center [2015-2017]: Dr. Herbert L. Bonkovsky (PI)

University of Michigan Ann Arbor [2003-2017]: Drs. Robert Fontana (PI), Hari Conjeevaram and Richard Moseley.

University of California, San Francisco, California Pacific Medical Center [2003-2013]: Drs. Timothy Davern (PI), and Mauricio Bonacini

Indiana University-Purdue [2003-2017]: Drs. Naga Chalasani (PI), Raj Vuppalanchi, Marwan Ghabril

Icahn School of Medicine at Mount Sinai [2013-2017]: Drs. Joseph A. Odin (PI), Jawad Ahmad (co-PI),

Mayo Clinic College of Medicine [2009-2013]: Dr. Jay Talwalkar (PI).

University of Pennsylvania [2009-2013]: Dr. Raj Reddy (PI).

University of Southern California and University of California-Los Angeles [2009-2017]: Drs. Andrew Stolz (PI), Neil Kaplowitz, Francisco Durazo

Albert Einstein Medical Center and University of Pennsylvania [2008-2017]: Dr. Vic Navarro (PI), Raj Reddy.

University of Texas-Southwestern [2008-2013]: Drs. William Lee, Lafaine Grant, and Donald Rockey.
