# Supplementary material for: Cytokine profiles in acute liver injury—Results from the US Drug-Induced Liver Injury Network (DILIN) and the Acute Liver Failure Study Group
Source: PLoS One. 2018 Oct 25;13(10):e0206389. doi: 10.1371/journal.pone.0206389 (PMC6201986; doi:10.1371/journal.pone.0206389)
Supplement: S2 Table — (DOCX) [file pone.0206389.s002.docx]

**S Table 2. Causative Agents of Acute Drug-Induced Liver Injury among Subjects from the DILIN**

| Isoniazid | 10 | Phenytoin | 2 | Erythromycin W/Sulfisoxazole | 1 |
| --- | --- | --- | --- | --- | --- |
| Sulfamethoxazole W/Trimethoprim | 10 | Propylthiouracil | 2 | Exemestane | 1 |
| Amoxicillin W/Clavulanic Acid | 9 | Quetiapine | 2 | Gabapentin | 1 |
| Unspecified Herbal | 6 | Valproic Acid | 2 | Herbal Nos W/Minerals Nos/Vitamins Nos | 1 |
| Ciprofloxacin | 5 | Amino Acids Nos | 1 | Hydroxycut - Ephedra Free | 1 |
| Anabolic Agents For Systemic Use | 4 | Amoxicillin | 1 | Imetelstat | 1 |
| Levofloxacin | 4 | Anakinra | 1 | Ipilimumab | 1 |
| Azithromycin | 3 | Antithymocyte Immunoglobulin | 1 | Linezolid | 1 |
| Mercaptopurine | 3 | Asparaginase | 1 | Lisinopril | 1 |
| Minocycline | 3 | Azathioprine | 1 | Micafungin | 1 |
| All Other Therapeutic Products | 2 | Bupropion | 1 | Montelukast | 1 |
| Amiodarone | 2 | *Camellia Sinensis* | 1 | Nefazodone | 1 |
| Antiinflammatory And Antirheumatic Products, | 2 | Carbohydrates/Proteins/Minerals/Vitamins, Com | 1 | Nitrofurantoin | 1 |
| Carbamazepine | 2 | Cefalexin | 1 | Other Combinations Of Nutrients/HDS | 1 |
| Clarithromycin | 2 | Cefazolin | 1 | Oxacillin | 1 |
| Diclofenac | 2 | Cefotaxime | 1 | Piperacillin Sodium W/Tazobactam | 1 |
| Escitalopram | 2 | Ceftriaxone | 1 | Rosuvastatin | 1 |
