# Supplementary material for: Cytokine profiles in acute liver injury—Results from the US Drug-Induced Liver Injury Network (DILIN) and the Acute Liver Failure Study Group
Source: PLoS One. 2018 Oct 25;13(10):e0206389. doi: 10.1371/journal.pone.0206389 (PMC6201986; doi:10.1371/journal.pone.0206389)
Supplement: S3 Table — (DOCX) [file pone.0206389.s003.docx]

**S Table 3. Drug causes of Acute**

**Drug-Induced Liver Injury among Subjects from the Acute Liver Failure Registry**

Herbals & Supplements 2

Isoniazid [INH] 3

Cerivastatin 2

Piperacillin-tazobactam 2

Interferon-beta 1

Isoflurane 1

INH+rifampicin 1

MDMA 1

Pravastatin 1

Propylthiouracil 1

Quetiapine 1

Trimethoprim-sulfamethoxazole 3

Nitrofurantoin 3

6-mercaptopurine 1

Amoxicillin-clavulanate 1

Anabolic steroid 1

Atorvastatin 1

Bromfenac 1

Carbamazepine 1

Ciprofloxacin 1

Didanosine 1

Disulfiram 1

Doxycycline 1

Fluconazole 1

Gemtuzumab 1

Abbreviations: MDMA, 3,4-methylenedioxy-methamphetqmine [aka Ecstasy or Molly]
