# Supplementary material for: Cytokine profiles in acute liver injury—Results from the US Drug-Induced Liver Injury Network (DILIN) and the Acute Liver Failure Study Group
Source: PLoS One. 2018 Oct 25;13(10):e0206389. doi: 10.1371/journal.pone.0206389 (PMC6201986; doi:10.1371/journal.pone.0206389)
Supplement: S4 Table — (PDF) [file pone.0206389.s004.pdf]

S4 Table. Causative Agents of Acute Drug-Induced Liver Injury among Subjects from the Acute Liver Failure Registry

Herbals & Supplements	2
Isoniazid [INH]	3
Cerivastatin	2
Piperacillin-tazobactam	2
Interferon-beta	1
Isoflurane	1
INH+rifampicin	1
MDMA	1
Pravastatin	1
Propylthiouracil	1
Quetiapine	1
Trimethoprim-sulfamethoxazole	3
Nitrofurantoin	3
6-mercaptopurine	1
Amoxicillin-clavulanate	1
Anabolic steroid	1
Atorvastatin	1
Bromfenac	1
Carbamazepine	1
Ciprofloxacin	1
Didanosine	1
Disulfiram	1
Doxycycline	1
Fluconazole	1
Gemtuzumab	1

Abbreviations: MDMA, 3,4-methylenedioxy-methamphetamine [aka Ecstasy or Molly]
